# Hunger extends lifespan by modulating histone proteins

**DOI:** 10.1093/lifemeta/load024

**Published:** 2023-06-12

**Authors:** Hailan Liu, Hongjie Li, Yong Xu

**Affiliations:** USDA/ARS Children’s Nutrition Research Center, Department of Pediatrics, Baylor College of Medicine, Houston, TX 77030, United States; Department of Molecular and Human Genetics, Baylor College of Medicine, Houston, TX 77030, United States; Huffington Center on Aging, Baylor College of Medicine, Houston, TX 77030, United States; USDA/ARS Children’s Nutrition Research Center, Department of Pediatrics, Baylor College of Medicine, Houston, TX 77030, United States; Department of Molecular and Cellular Biology, Baylor College of Medicine, Houston, TX 77030, United States; Section of Endocrinology, Diabetes, and Metabolism, Department of Medicine, Baylor College of Medicine, Houston, TX 77030, United States

**In a recent study published in Science, Weaver *et al*. provided insights into the effects of hunger on longevity. Inducing a state of hunger, either through restricting isoleucine intake or by stimulating R50H05 hunger neurons, resulted in an extension of lifespan in fruit flies. This effect is mediated by the modulation of histone proteins in the brain.**

Extensive research conducted since the early 1900s has focused on understanding the effects of reducing food intake on preventing age-related diseases and increasing the lifespan of fruit flies, mice, and rats [1], highlighting the potential of calorie restriction (CR) as a promising intervention for extending lifespan. However, recent evidence has emphasized the significance of dietary composition, rather than total calorie intake, as the key determinant of the longevity effects of CR [2]. In support of this notion, studies have shown that restricting the intake of specific nutrients, such as methionine alone, is sufficient to increase the lifespan of various organisms including yeast, fruit flies, and rodents [3]. Among these nutrients, branched-chain amino acids (BCAAs) play important roles in health and longevity. High BCAA levels have been associated with insulin resistance, diabetes, and an increased risk of age-related diseases. Conversely, low BCAA intake improves glucose homeostasis and extends lifespan in mice [4, 5].

In a recent study published in Science [6], Weaver *et al*. reported that low-BCAA diets increase feeding and survival, and the motivational state of hunger alone can promote longevity in fruit flies. To specifically investigate the effects of BCAAs on food intake and lifespan, the authors designed a reference holidic diet that allowed them to adjust the concentration of BCAAs without altering other dietary components. Compared to flies fed on high-BCAA diets, those fed on low-BCAA diets consumed more food but lived significantly longer, suggesting that restricting BCAA consumption promotes longevity despite hyperphagia. To determine the impact of BCAAs on hunger states, the researchers subjected food-deprived flies to low- or high-BCAA diets. Flies refeeding on a low-BCAA diet ingested significantly more food than those refeeding on a high-BCAA diet, indicating that BCAA restriction elevated the state of hunger. The BCAAs are composed of three different amino acids: leucine, isoleucine, and valine. Further investigations revealed that decreasing isoleucine, but not leucine or valine, was required to increase feeding. Replenishing isoleucine to low-BCAA diets attenuated food intake, demonstrating that isoleucine acts as a dietary signal to modulate feeding.

Remarkably, reducing isoleucine intake exaggerated protein appetite and extended lifespan to a similar degree as reducing all BCAAs. This led the researchers to explore whether hunger itself could slow aging. To this end, R50H05 hunger neurons were optogenetically activated to induce a heightened state of hunger. Stimulating R50H05 neurons promoted longevity, suggesting that hunger itself can enhance survival and increase lifespan.

Moving forward, the authors focused on the alterations in histone proteins to unravel the molecular mechanisms underlying hunger-induced longevity. Low-BCAA diets significantly decreased the abundance of histone H3 and its acetylation at the H3K9 site in the brain, such effects were abrogated by feeding flies with isoleucine or the histone deacetylase inhibitor, sodium butyrate. Similarly, activating R50H05 neurons reduced H3 abundance in fly heads.

To further validate the role of histone acetylation in regulating feeding and lifespan, flies were fed with sodium butyrate for one week. Inhibiting histone acetylation abolished the increased feeding, as well as longevity, observed on low-BCAA diets. Furthermore, removing the histone deacetylase *Rpd3* using the loss-of-function mutant *Rpd3*^def24^ extended the lifespan of flies on high-BCAA food. Together, these findings suggest that histone modifications are required to mediate the beneficial effects of low-BCAA diets on aging.

BCAAs are metabolized by branched-chain amino transferase (BCAT) in target tissues [7]. When *BCAT* was selectively deleted in R50H05 neurons through an RNAi-mediated approach, the effects of low-BCAA diets on reducing histone H3 and prolonging lifespan were eliminated, although feeding behavior was unaffected. Interestingly, *BCAT* ablation in serotonergic neurons decreased feeding and prevented overeating on low-BCAA diets. Thus, distinct neural populations may mediate the influences of BCAAs on feeding and longevity.

In summary, the study by Weaver *et al*. shed fascinating light on the effects of hunger on longevity. Manipulating the hunger state, either through restricting isoleucine intake or by stimulating R50H05 hunger neurons, resulted in an extension of lifespan in fruit flies. Particularly, this effect was attributed to the modulation of histone proteins in the brain.

One recent study showed that CR can increase the mouse lifespan by 10%, and daily fasting on dietary food aligned with the circadian cycle can extend lifespan by 35% [8]. This finding suggests that fasting and circadian interventions can enhance the lifespan benefits of caloric restriction. It is also noteworthy that previous research has highlighted the influences of nutritional status on histone protein expression in rodents [9]. Given these connections, it would be intriguing to investigate whether the induction of hunger sensations, such as by activating the well-known orexigenic Agouti-related protein neurons, in other species could similarly regulate histone abundance and potentially enhance longevity. Moreover, there is a reciprocal relationship between hunger and BCAAs in mice. On one hand, food deprivation elevates plasma BCAAs. On the other hand, BCAAs have been shown to suppress feeding [10]. The intricate relationship between hunger, BCAAs, and longevity in mammals remains to be elucidated. Further, future studies exploring the specific roles of various neural circuits in mediating the effects of BCAAs could provide valuable insights into the underlying mechanisms and potentially uncover new therapeutic avenues for modulating feeding behavior and promoting healthy aging.
